# Global research trends and hotspots in metabolomics of osteosarcoma: a decade-spanning bibliometric and visualized analysis

**DOI:** 10.3389/fimmu.2024.1463078

**Published:** 2024-10-09

**Authors:** Jun-Bo Tu, Tao Liu, Jun-Feng Li, Jian Long, Xiu Wang, Wen-Cai Liu, Xing-Hua Gao

**Affiliations:** ^1^ Department of Orthopaedics, Guangzhou First People’s Hospital, South China University of Technology, Guangzhou, China; ^2^ Department of Oethopaedics, Xinfeng County People's Hospital, Ganzhou, Jiangxi, China; ^3^ Department of Clinical Medicine, Fuzhou Medical College of Nanchang University, Fuzhou, Jiangxi, China; ^4^ Department of Orthopaedics, Shanghai Sixth People’s Hospital Affiliated to Shanghai Jiao Tong University School of Medicine, Shanghai, China

**Keywords:** osteosarcoma, metabolism, research hots, Web of Science, publication volume

## Abstract

**Object:**

Osteosarcoma is a malignant tumor originating from the bones, commonly found in children and adolescents, especially in rapidly growing bone areas such as the knees and upper arms. In this study, we aim to delineate the evolution and convergence of research themes in osteosarcoma metabolomics over the past decade, identify major contributors, and forecast emerging trends that could direct future research efforts.

**Method:**

The bibliometric method has been applied to systematically analyze the literature in the field of osteosarcoma metabolomics. The relevant literatures were collected from the Web of Science Core Collection, spanning from January 1, 2014, to December 31, 2023. Tools such as CiteSpace, Bibliometrix, and VOSviewer were used for the visual analysis of the collected literatures. The focused information includes institutions, journals, countries, authors, keywords, and citations.

**Result:**

Various aspects in the field of osteosarcoma metabolism were analyzed. Shanghai Jiao Tong University has published the most papers in the past ten years, followed by Central South University and Zhejiang University. Among the sources, the international journal of molecular sciences publishes the most articles, and oncotarget is the journal with the highest H index. According to Bradford’s law, there are 34 core journals identified. A total of 5501 authors participated in the creation of papers in this field. The distribution of authors follows Lotka`s Law, and 85.3% of authors have only one article. 46% of the corresponding authors are from China, but most of these corresponding authors are not good at international cooperation. China also has the largest number of publications, followed by the United States. It can be confirmed that China dominates this field. Among the keywords, “expression” is the keyword that has received the most attention in the past ten years. All keywords can be divided into 9 clusters. Based on the explosive words and hot topics each year, we speculate that future research will focus on the tumor microenvironment, molecular mechanisms and autophagy, targeted therapies and inhibitors.

**Conclusion:**

In summary, this study comprehensively analyzed the current state of research in the field of osteosarcoma metabolism through bibliometric methods. The findings revealed the development trends and research hotspots in this field, which may provide valuable references for future research directions.

## Introduction

Osteosarcoma is a malignant tumor originating in the bones, commonly affecting children and adolescents, particularly in rapidly growing skeletal regions like the knees and upper arms (1). It is characterized by high heterogeneity and aggressiveness, often accompanied by early lung metastasis. Treatment typically includes surgical resection and chemotherapy (2).

Metabolomics, as a branch of systems biology, is dedicated to the comprehensive analysis of metabolites in organisms. In the study of osteosarcoma, metabolomics provides a new perspective for understanding tumor treatment (3). By analyzing metabolite differences between osteosarcoma cells and normal bone cells, researchers were able to reveal how tumor growth, metastasis, and response to treatment are linked to metabolic processes. For example, studies on energy metabolism have found that osteosarcoma cells favor glycolysis over oxidative phosphorylation (4). Research into amino acid metabolism has found that abnormally high levels of glutamate may be related to osteosarcoma’s ability to proliferate and survive (5). Studies of lipid metabolism have found that the concentrations of specific lipid molecules in osteosarcoma tissue are significantly different from normal bone tissue (6).

Research into the metabolism of osteosarcoma not only helps in understanding the development and progression of the disease but also holds potential for developing new treatment strategies in the future. Moreover, monitoring specific metabolic products can provide valuable insights for personalized treatment plans, tailoring therapies based on the unique metabolic profile of each patient’s tumor (7).

Bibliometric analysis is an internationally recognized method of information processing. It not only assists governments in making important budgetary decisions (8) but also helps in understanding emerging fields of study in rapidly developing areas.

This study systematically analyzes the collected data on institutions, journals, authors, countries or regions, keywords, and cited references using bibliometric methods. The software tools utilized include Cite Space, VOSviewer and R language. These tools enable performance analysis and visualization of the data, providing predictive references for future research directions.

Cite Space is an open-source software tool for bibliometric and visual analysis, designed to help researchers explore relationships in academic literature, research trends, and collaboration networks (9, 10). Developed by Professor Chaomei Chen of Drexel University, it offers robust features that aid in better understanding literature data.

Bibliometrix is a program package (www.bibliometrix.org) developed based on the r language, which provides a range of tools and features for bibliometric analysis and scientometric analysis (11). Benefiting from the powerful statistical analysis and graphical representation capabilities of the r language, Bibliometrix is capable of performing a wide range of complex analytical tasks, such as author collaboration network analysis, co-citation analysis, time series analysis, keyword rendering, and so on. With this package, users can easily perform statistical analysis and visualization of collected data.

VOSviewer is a software tool developed by Nees Jan van Eck and Ludo Waltman at Leiden University’s Centre for Science and Technology Studies in the Netherlands. It is specifically designed to construct and visualize bibliometric networks. These networks can include journals, researchers, or individual articles, which can be linked by citations, co-citations, bibliographic couplings, or co-authorship relations (12). The primary function of VOSviewer is to visually explore these networks to better understand the relationships and major trends within a specific scientific domain. Its intuitive interface allows users to effectively map and examine the intricate network of scientific publications, facilitating a deeper insight into the landscape of academic research.

As time progresses, more research is being conducted in the field of osteosarcoma metabolism, but currently, there is no study that systematically analyzes the status of this field. This study was guided by a fundamental research question: How have research topics in the field of osteosarcoma metabolism evolved over the past decade? Who are the major contributors in this field? Focusing on these two questions, We conducted this research. By delving into this issue, we aim to shed light on the evolution of themes in this field and identify the main contributors. This question not only allows us to chart the historical trajectory of metabolomic research in osteosarcoma but also to predict emerging trends and propose a roadmap for future research efforts.

## Methods

### Search strategies and data acquisition methods

**Figure 1:**
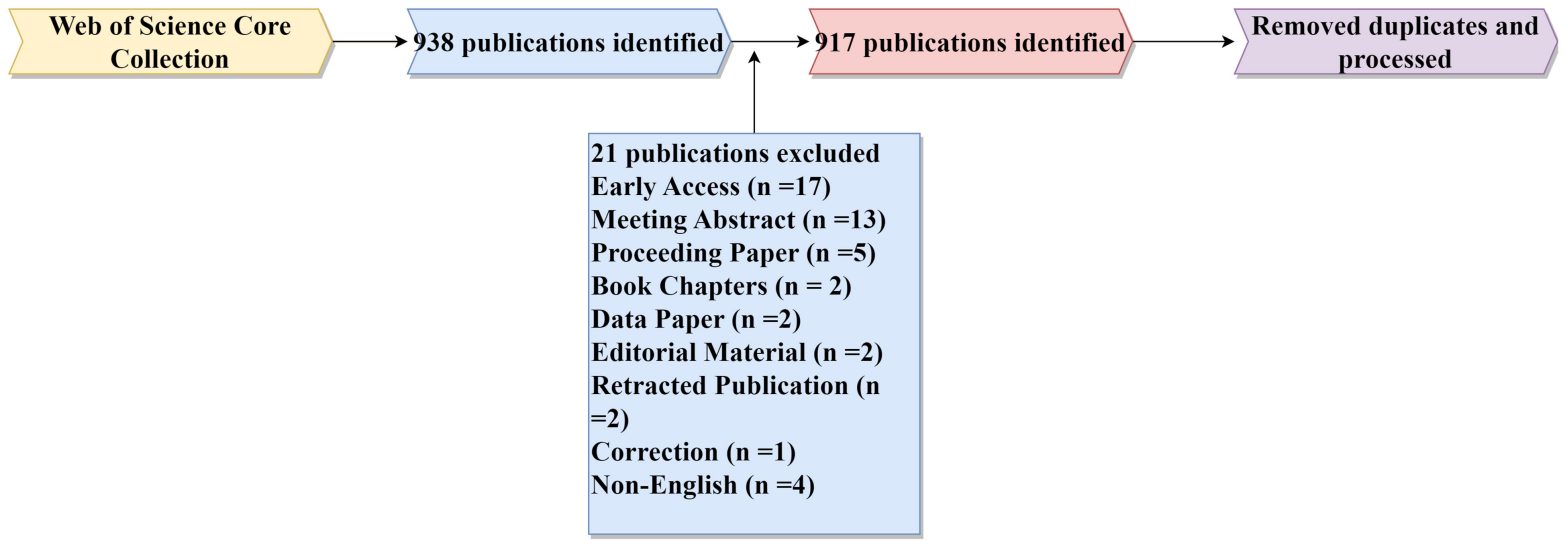
The rough process of this experiment.

The data for this study was collected from the Web of Science Core Collection(WoSCC), which is considered a high-quality literature repository and is widely recognized by scholars (13). Considering the need to exclude some less important papers, we decided to collect literature from only one database, WoSCC. The search formula “TS = (osteosarcoma and metaboli*)” was entered in the advanced search bar of WoSCC, with the time frame set from January 1, 2014, to December 31, 2023. A total of 938 search results were included. Metaboli* contains all terms related to metabolism, and it serves our purpose well by expanding the scope of the search as much as possible but not retrieving beyond metabolism. The collection of data was done by J. Long and J-F. Li, followed by a screening session by J. Long, J-F. Li and X. Wang, with X. Wang judging and deciding when there was a difference of opinion in the screening. Through a screening process, excluding certain types with few publications like “early access,” “meeting abstract,” and “data paper.” Non-English literature was also removed due to its rarity and the current inability of analysis software to process non-English languages. Ultimately, 917 publications were selected for this study. This data includes institutions, journals, authors, countries or regions, keywords, titles, and cited references. The access and search strategy is illustrated in **Figure 1**. The search results were exported by clicking ‘export’, selecting ‘plain text file’, and ‘full record and cited references’.

### Data analysis

Rename the filtered data as “download” and import it into citespace (cite space 6.3.R1): select “import/export” under “Data” to remove duplicates. Save the results separately. Select “New” to create a new project, and select the data path to save the results of the previous step. “Time Slicing” is “From 2014 JAN to 2023 DEC”, “Years Per Slice” option 2. And keeping the defaults for the other projects, and select the corresponding projects in Node Types for the visual analysis.

Select “Create” in the main page of VOSviewer, choose “Create a map based on bibliographic data” in “Choose type of data” and click “Next”. Then select “Read data form bibliographic database files” in “Choose data source” and click “Next”. Under “Web of Science”, select the path to save the filtering results. Under “Fields from which terms will be extracted”, select “Title and abstract fields” and click “Next”. In “Choose type of analysis and counting method”, select “Co authorship”, “Authors” and “Full counting” and click “Finish”. Under the main page, perform the corresponding visualization and analysis (leave the default settings unmentioned).

With the Bibliometrix package loaded in R language, users can directly import data and run analyses, resulting in comprehensive visual analytics.

## Result

### Overview of publications

**Figure 2:**
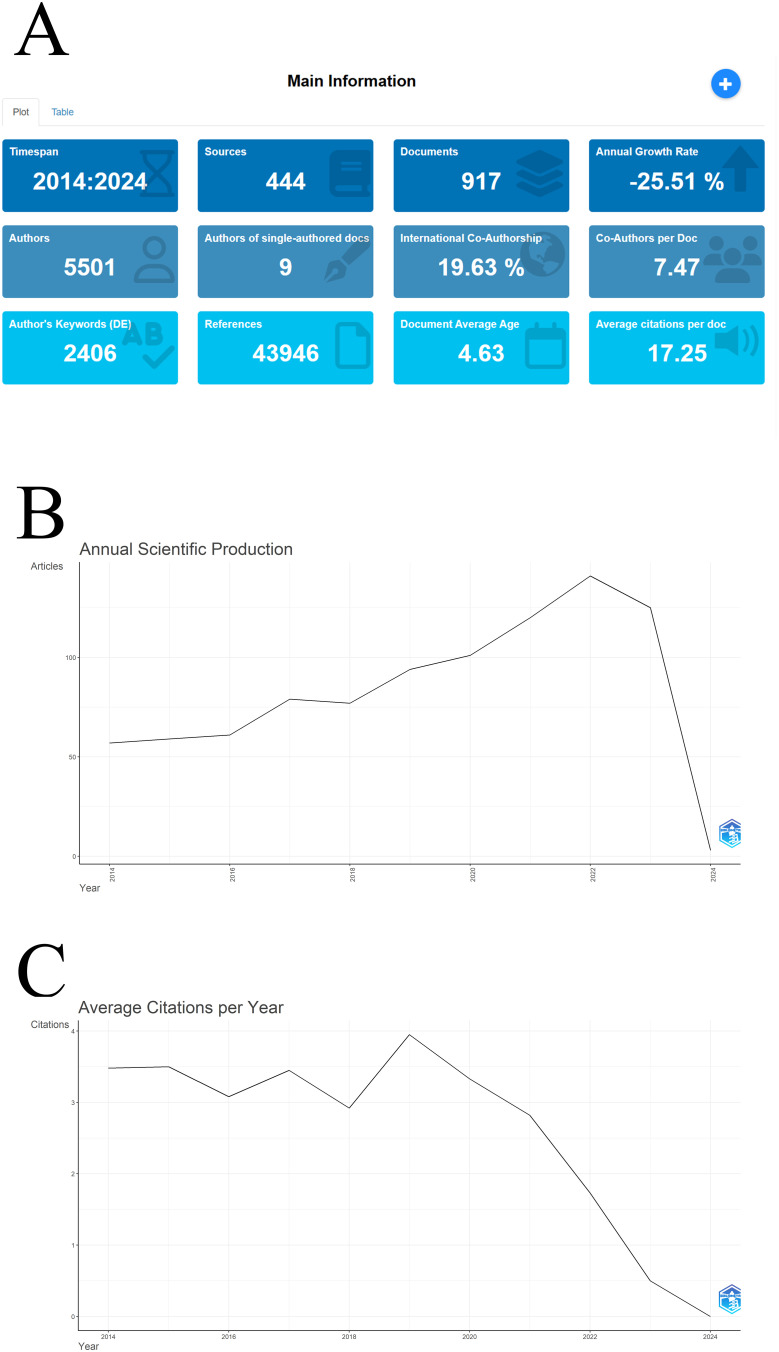
The general situation of publications in this field: **(A)** Main information; **(B)** Annual scientific product map; **(C)** Average citation frequency in each year.

This study includes literature submitted in 2023, expected to be published in 2024. This inclusion causes Bibliometrix to account for 2024 in its analysis, but to maintain the integrity of the 2023 research, these documents are retained. **Figure 2A** shows the overall situation of the collected literature, such as the time span, number of sources, and number of authors. **Figure 2B** illustrates the annual scientific output in this field, showing an upward trend in publications until 2022, followed by a decline. **Figure 2C** depicts the annual average citations of these articles, showing fluctuations before 2019 but mostly ranging between 3-4, and a declining trend afterward, with 2019 being a peak. This suggests that significant research addressing key issues was published in 2019, after which the focus of research shifted away from mainstream attention. **Table 1** lists the top ten most cited papers, with the most cited being a 2015 paper by PIRES RA et al. on new cancer treatment methods, widely tested in osteosarcoma (14). The paper with the highest standardized citation frequency is by FU JK et al., published in 2021, discussing solutions to drug resistance in solid tumors, contributing to the effectiveness of chemotherapy (15).

**Table 1:** The list of top ten most cited papers.

Paper	DOI	Total Citations	TC per Year	Normalized TC
PIRES RA, 2015, J AM CHEM SOC	10.1021/ja5111893	226	22.60	6.45
WONG AST, 2015, NAT PROD REP	10.1039/c4np00080c	202	20.20	5.76
GOLD ES, 2014, P NATL ACAD SCI USA	10.1073/pnas.1404271111	177	16.09	4.62
BRÖER A, 2016, J BIOL CHEM	10.1074/jbc.M115.700534	156	17.33	5.63
SAMPSON VB, 2015, FRONT PEDIATR	10.3389/fped.2015.00069	154	15.40	4.39
PAPPO AS, 2014, CANCER-AM CANCER SOC	10.1002/cncr.28728	142	12.91	3.71
SONG JY, 2017, BIOCHEM BIOPH RES CO	10.1016/j.bbrc.2017.06.024	132	16.50	4.78
XIN M, 2016, ONCOTARGET	10.18632/oncotarget.10020	130	14.44	4.70
FU JK, 2021, BIOMATERIALS	10.1016/j.biomaterials.2020.120537	125	31.25	11.09
SARKAR N, 2019, ACS APPL MATER INTER	10.1021/acsami.9b01218	119	19.83	5.02

### Institution analysis

**Figure 3:**
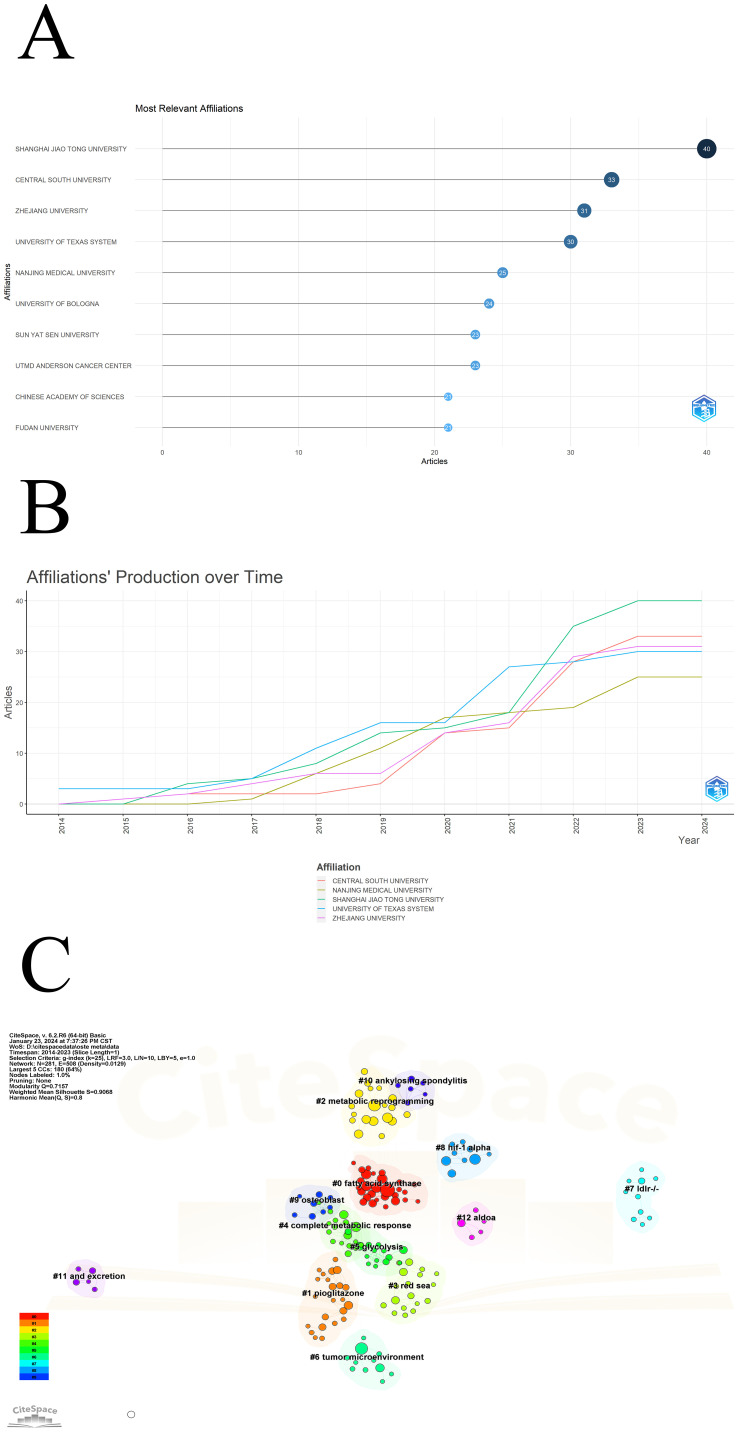
Institutional information: **(A)** Ranking of institutions by publication volume; **(B)** Annual scientific output of the top five institutions by publication volume; **(C)** Clustering of institutions by keywords.

The volume of publications by research institutions can reflect their interest and research capabilities in a particular field. In the field of osteosarcoma metabolism, the institution with the most publications is Shanghai Jiao Tong University (16), followed by Central South University (17), and Zhejiang University (18), as shown in **Figure 3A**. The top ten publishing institutions are listed in **Figure 3A**. The relationship between their publications and time is depicted in **Figure 3B**, indicating an upward trend in publications from these institutions. Using the Cite Space clustering algorithm, these institutions have been clustered based on the keywords of their publications into 13 categories (**Figure 3C**).

1. Fatty Acid Synthase: Institutions under this cluster may wish to pursue therapeutic options through the role of fatty acid synthase in osteosarcoma. For example, some studies indicate that FASN plays a crucial role in the progression and metastasis of osteosarcoma, making it a promising target for therapeutic interventions (19, 20).
2. Pioglitazone: Several institutions are working on the effects of pioglitazone in osteosarcoma. The potential of pioglitazone as a resistance modulator and as a component of combination therapy in osteosarcoma points to more effective strategies to overcome chemoresistance in cancer treatment (21–23).
3. Metabolic Reprogramming: Some institutions are interested in Metabolic Reprogramming. Their studies collectively demonstrate the importance of metabolic pathways in the progression of osteosarcoma and suggest potential biomarkers and targets for therapeutic intervention (24–26).
4. Red Sea: The research of this part of the institution is based on the Red Sea Metagenomic Library, hoping to find ways to treat cancer from the genes of Red Sea microorganisms (27).
5. Complete Metabolic Response: These institutions hope to understand the complete metabolic process of osteosarcoma to provide new insights into the occurrence and development of osteosarcoma (28).
6. Glycolysis: The research center of these institutions is glycolysis, and they hope to find treatment options for osteosarcoma through the glycolysis pathway (4, 29).
7. Tumor Microenvironment: These institutions provide a broad overview of how the tumor microenvironment influences osteosarcoma development, progression, and response to treatment, offering insights into potential targeted therapies (30, 31).
8. LDLR -/-: Research at these institutions revolves around the low-density lipoprotein receptor, looking for its relationship with osteosarcoma (32).
9. HIF-1 Alpha: These institutions are committed to demonstrate the central role of HIF-1α in the progression, metastasis, and chemoresistance of osteosarcoma, making it a significant target for developing new therapeutic strategies (18, 33).
10. Osteoblast: These institutions provide insights into the complex interactions between osteosarcoma cells and osteoblasts, highlighting the potential for targeted therapeutic strategies based on these relationships (17).
11. Ankylosing Spondylitis: These institutions provide a broader understanding of the complex interactions between inflammatory processes and bone remodeling in ankylosing spondylitis, which could indirectly relate to the pathology of osteosarcoma (34, 35).
12. And Excretion: These institutions have delved into different aspects of excretion relevant to osteosarcoma, providing valuable data on bone metabolism and potential diagnostic biomarkers (36, 37).
13. These institutions are committed to finding A possible treatment for osteosarcoma in Aldolase A (38).

All institutions in this field can be roughly classified into these thirteen clusters, reflecting the complexity and multidisciplinarity of osteosarcoma metabolism research. Using Cite Space, we obtained a burst table (**Table 2**) for institutions, including 8 institutions. Some institutions, like Free University of Berlin, Charite Universitatsmedizin Berlin, and Humboldt University of Berlin, showed keen interest in this field between 2014-2016, notably all from Berlin, Germany. Additionally, two institutions, Zhengzhou University and Nanchang University from China, have increased their research in this field starting from 2021.

**Table 2:** Top 8 institutions with the strongest citation bursts.

Institutions	Year	Strength	Begin	End	2014 - 2023
Free University of Berlin	2014	3	2014	2016	▃▃▃▂▂▂▂▂▂▂
Charite Universitatsmedizin Berlin	2014	2.5	2014	2016	▃▃▃▂▂▂▂▂▂▂
Humboldt University of Berlin	2014	2.5	2014	2016	▃▃▃▂▂▂▂▂▂▂
Institut National de la Sante et de la Recherche Medicale (Inserm)	2015	2.58	2015	2016	▂▃▃▂▂▂▂▂▂▂
IRCCS Istituto Ortopedico Rizzoli	2016	2.47	2016	2017	▂▂▃▃▂▂▂▂▂▂
Nanjing Medical University	2018	3.45	2018	2020	▂▂▂▂▃▃▃▂▂▂
Zhengzhou University	2021	2.47	2021	2023	▂▂▂▂▂▂▂▃▃▃
Nanchang University	2020	2.45	2021	2023	▂▂▂▂▂▂▂▃▃▃

### Source analysis

**Figure 4:**
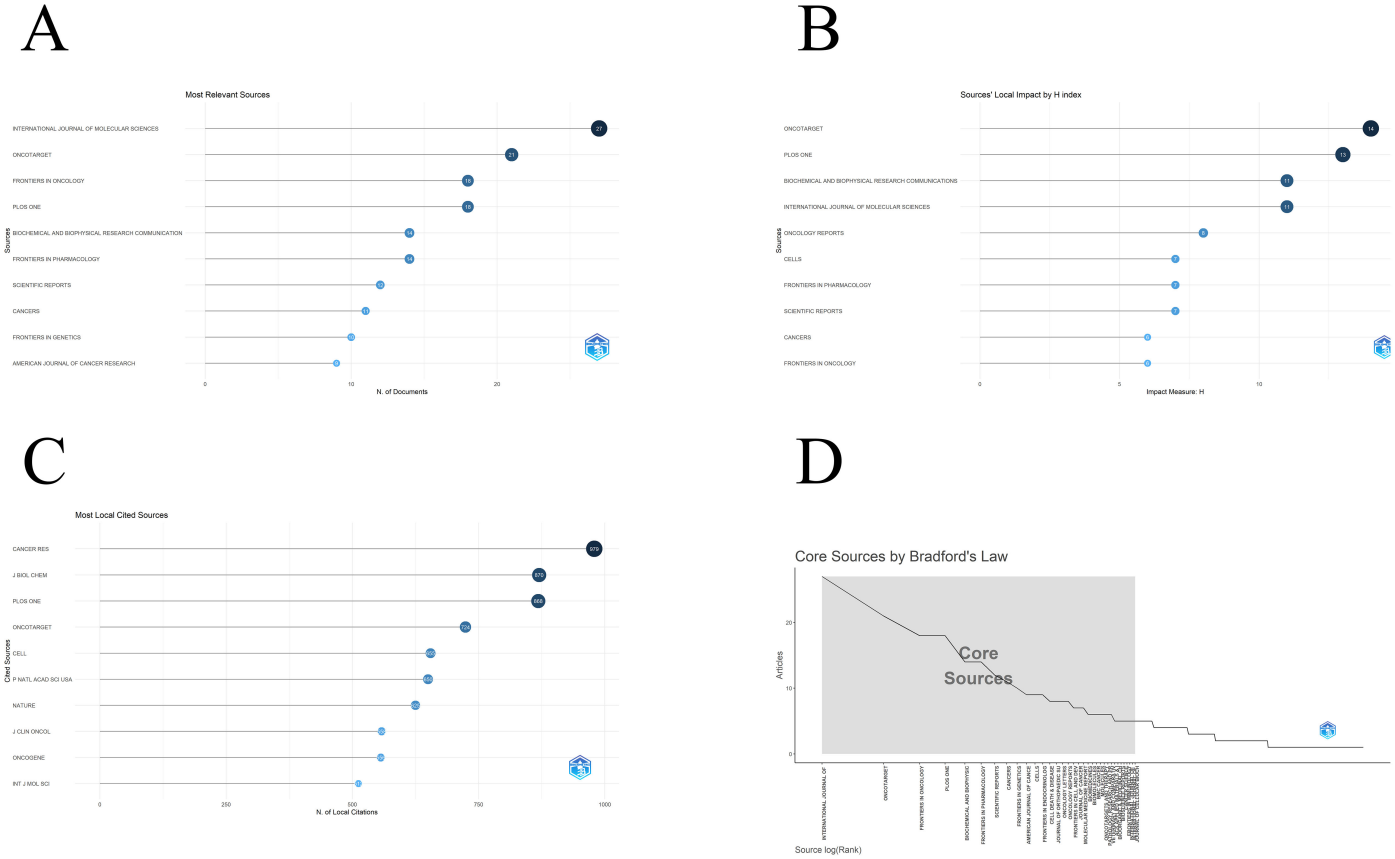
Journal information: **(A)** Journal publication volume ranking; **(B)** Journal H index ranking; **(C)** Journal citation ranking; **(D)** Core journal distribution.

For the analysis of journals, we extracted the number of publications, h-index and citation frequency as references. The top ten journals by publication volume are shown in **Figure 4A**. The top three are international journal of molecular sciences (29), oncotarget (24), Frontiers in oncology (21) and plos one (21). The top ten journals with H index are shown in **Figure 4B**. The top three are oncotarget (14), plos one (13), international journal of molecular sciences (11) and biochemical and biophysical research communications (11). Frontiers in oncology ranks tenth on the h-index list, which is a positive phenomenon. However, compared with other journals that are also in the top three publications, Frontiers in Oncology has a big gap. Perhaps frontiers in oncology should raise the inclusion threshold to include more valuable studies. Oncology Reports does not appear in the top ten by number of published articles, but its h-index ranks fifth, indicating that although this journal publishes fewer articles, its average quality is higher. The number of local citations can evaluate the influence of a journal in the industry. The journal with the most citations is cancer res (979), followed by j biol chem (870) and plos one (868). The remaining seven highly cited journals are shown in **Figure 4C**. Bradford’s law is a law used to evaluate the distribution of journals in a certain field. It is generally divided into three areas: core journals, secondary journals and tertiary journals. **Figure 4D** shows the distribution of journals in the field of osteosarcoma metabolism. The shaded area contains the core journals in this field. The specific list is shown in **Table 3**.

**Table 3:** Core journals in the field of osteosarcoma metabolomics.

SO	Rank	Freq	cumFreq
INTERNATIONAL JOURNAL OF MOLECULAR SCIENCES	1	27	27
ONCOTARGET	2	21	48
FRONTIERS IN ONCOLOGY	3	18	66
PLOS ONE	4	18	84
BIOCHEMICAL AND BIOPHYSICAL RESEARCH COMMUNICATIONS	5	14	98
FRONTIERS IN PHARMACOLOGY	6	14	112
SCIENTIFIC REPORTS	7	12	124
CANCERS	8	11	135
FRONTIERS IN GENETICS	9	10	145
AMERICAN JOURNAL OF CANCER RESEARCH	10	9	154
CELLS	11	9	163
FRONTIERS IN ENDOCRINOLOGY	12	9	172
CELL DEATH & DISEASE	13	8	180
JOURNAL OF ORTHOPAEDIC SURGERY AND RESEARCH	14	8	188
ONCOLOGY LETTERS	15	8	196
ONCOLOGY REPORTS	16	8	204
FRONTIERS IN CELL AND DEVELOPMENTAL BIOLOGY	17	7	211
JOURNAL OF CANCER	18	7	218
MOLECULAR MEDICINE REPORTS	19	7	225
BIOMEDICINES	20	6	231
BIOMOLECULES	21	6	237
BMC CANCER	22	6	243
MOLECULES	23	6	249
ONCOTARGETS AND THERAPY	24	6	255
PATHOLOGY RESEARCH AND PRACTICE	25	6	261
VETERINARY AND COMPARATIVE ONCOLOGY	26	6	267
ACS APPLIED MATERIALS & INTERFACES	27	5	272
BIOORGANIC & MEDICINAL CHEMISTRY LETTERS	28	5	277
BIOSCIENCE REPORTS	29	5	282
CANCER SCIENCE	30	5	287
FRONTIERS IN IMMUNOLOGY	31	5	292
INTERNATIONAL JOURNAL OF BIOLOGICAL SCIENCES	32	5	297
INTERNATIONAL JOURNAL OF ONCOLOGY	33	5	302
JOURNAL OF CELLULAR BIOCHEMISTRY	34	5	307

### Author analysis

**Figure 5:**
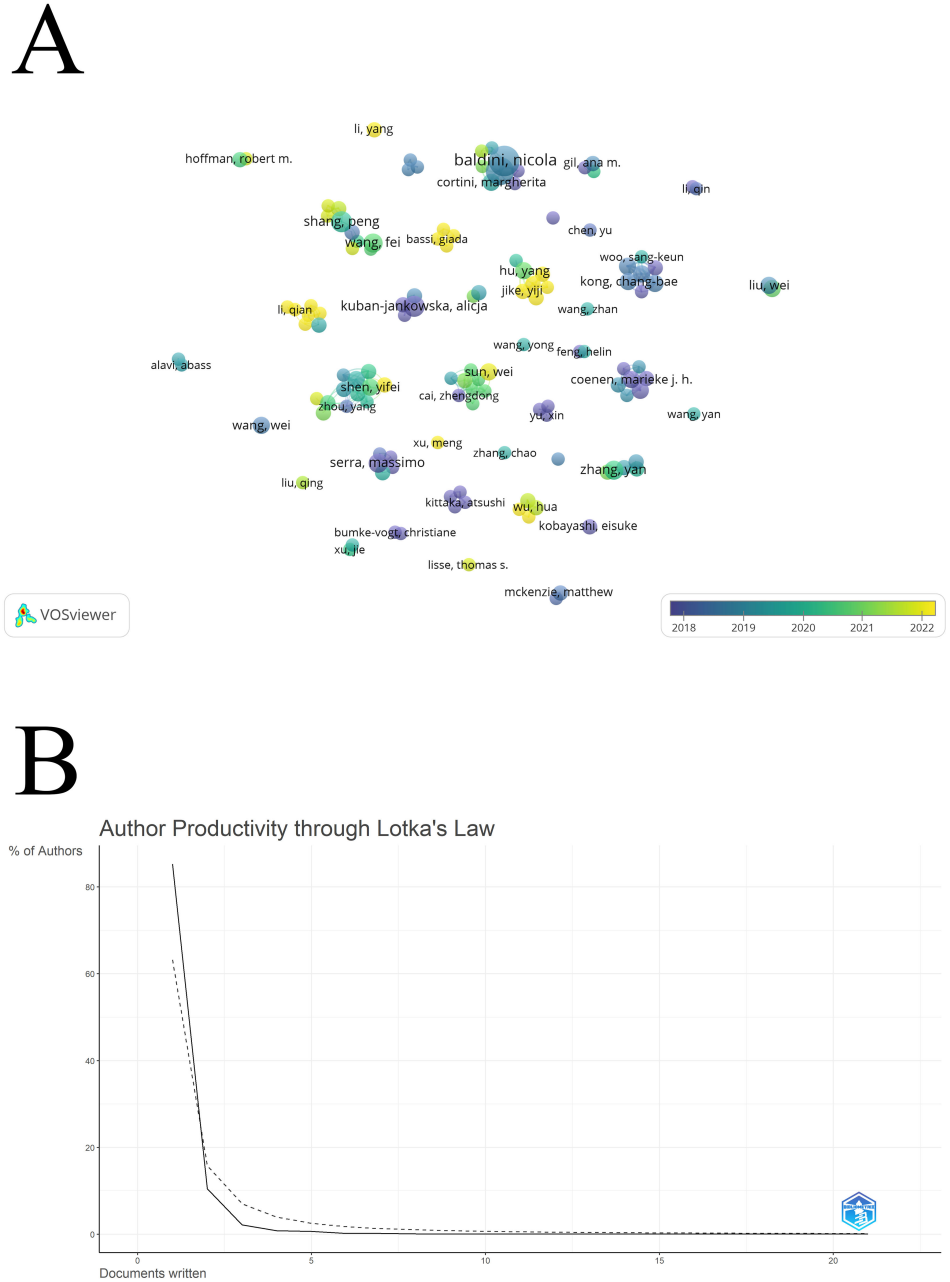
Author information: **(A)** Author collaboration network; **(B)** Author distribution by output.

The author collaboration network displays authors who have published 3 or more articles, as shown in **Figure 5A**. The authors in the figure are divided into several clusters, and the authors in each cluster have a close cooperation relationship. For example, the groups represented by Zhou, Yang and Shen, Yifei have close cooperation. The size of the label represents the author’s relative publication volume. For example, Baldini, Nicola, Kuban-Jankowska, and Alicja have more publications. We have collected the number of articles published by some authors who have detailed information in WOSCC (the authors of some articles have only one article and their initials are the same, but these authors have not claimed the article in WOS), and they are displayed in **Table 4**. The color of the node represents the year in which the author was active, and the year corresponding to the color is shown as the label in the lower right corner of the figure. A total of 5501 authors participated in this field in the past ten years. Most of these authors published only one article, which is similar to Lotka`s Law that describes the distribution of authors’ publications. **Figure 5B** compares the distribution of authors in this field (solid line) with Lotka`s Law (dashed line). Lotka’s Law was introduced by Alfred Lotka in 1926 and is used primarily to describe the distribution of scholarly output by scientists, authors, or artists (39). The approximate meaning of this law is that a very small number of people create most of the work, while the majority of people contribute only a very small portion. **Table 5** shows the authors with different numbers of publications. 85.3% of the authors published only one article, indicating that a large number of new authors have poured into this field in the past ten years, but the investment of these authors still needs to increase to publish more valuable research. It should be pointed out that some documents are attributed to one author because they are not claimed by the author, and he has published more articles than the author with detailed attribution.

**Table 4:** The number of articles published by authors who have detailed information in WOSCC.

Authors	Articles
Avbet, Soflia	12
Baldini, Nicola	10
Kuban-Jankowska, Alicja	7
Shang, Peng	6
Serra, Massimo	6
Cortini, Margherita	6
Shen,Yifei	6
Lim, Ilhan	5
Scotlandi,Katia	5
Song,Wonseok	5
Zhou, Dong	5
Gorska-Ponikowska,Magdalena	5
Guchelaar,henk-jan	5
Kong, Chang-Bae	5
Lim,Sang Moo	5
Byun, Byung Hyun	5
Lv,Huanhuan	5
Hua,Yingqi	4
Coenen,Marieke	4
Kobayashi, Eisuke	4

**Table 5:** Distribution of authors by number of publications.

Documents written	N. of Authors	Proportion of Authors
1	4693	0.853
2	575	0.105
3	118	0.021
4	44	0.008
5	37	0.007
6	9	0.002
7	11	0.002
8	3	0.001
9	2	0
10	3	0.001
11	1	0
12	1	0
13	1	0
14	1	0
18	1	0
21	1	0

### Country or region situation analysis

**Figure 6:**
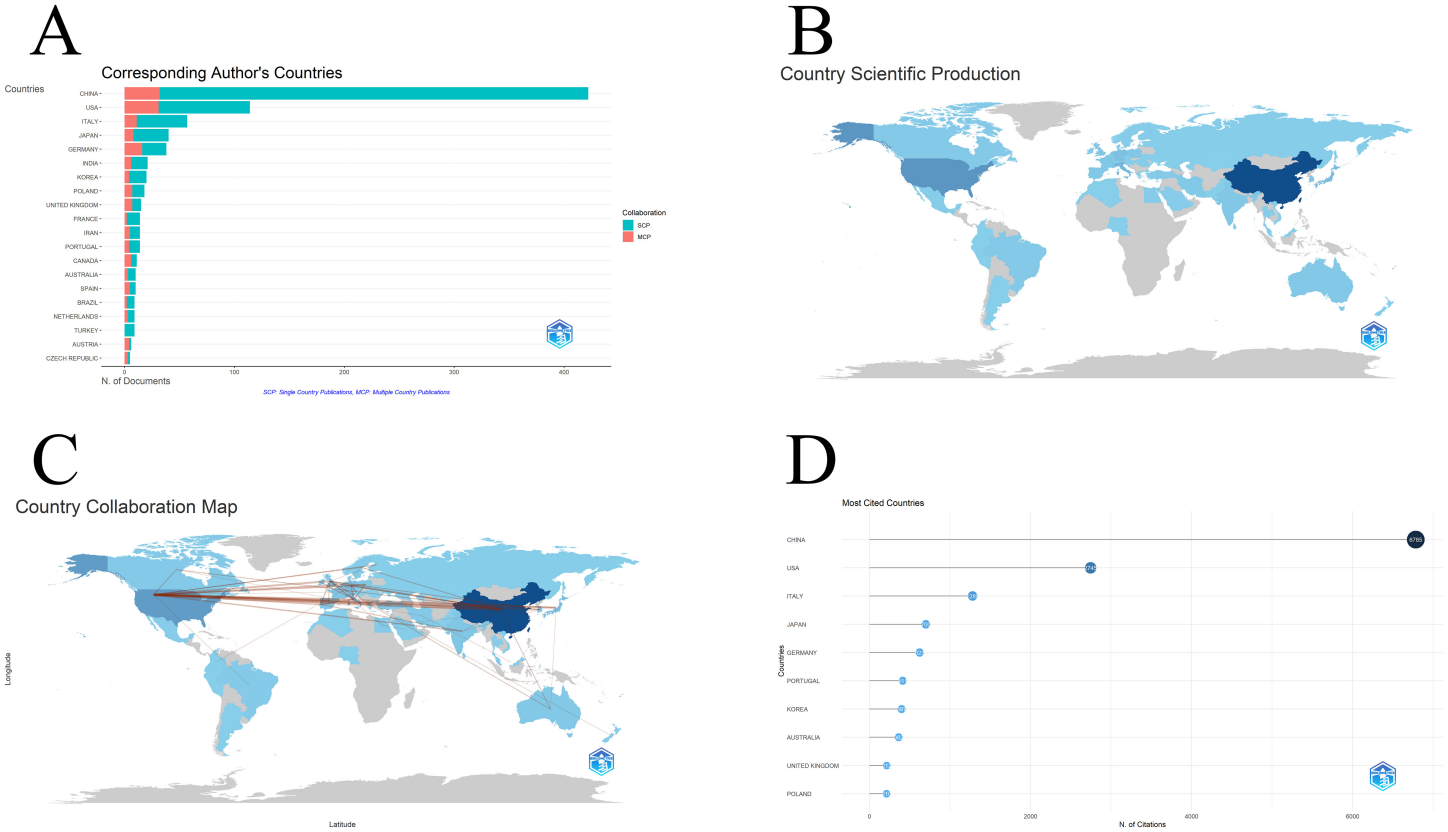
Country or region information: **(A)** The source of the corresponding author; **(B)** The status of scientific products in each country or region; **(C)** The cooperative relationship between countries or regions; **(D)** The citation status of each country or region.

We first determined the country of the corresponding author in the collected documents (**Figure 6A**). The largest number of corresponding authors are from China, accounting for 46% of the total corresponding authors (**Table 6**), followed by the United States and Italy. In the figure, MCP represents the corresponding author of international cooperation, and SCP represents the corresponding author of domestic cooperation. It is obvious that most of the corresponding authors in China cooperate with domestic personnel, and their international cooperation relationships are relatively weak. The United States has the close number of MCPs as China. Since the total number of people is smaller than that of China, the proportion of MCPs is larger. The situation in Italy is similar to that in China. Of course, there are also a small number of countries that are good at cooperating with other countries, such as Germany and the United Kingdom. Their MCP Ratio is greater than 40%. The details are shown in **Table 6**. **Figure 6B** shows the number of publications of each country or region in the form of a map, with regional colors. The depth of the represents the amount of articles published in the region. China has the darkest color in the picture, which means it has the largest amount of articles published, followed by the United States. All countries and regions are roughly divided into four levels. China and the United States are level one and level two respectively; countries with lighter colors than the United States, such as Germany, the United Kingdom, and Italy, are level three; countries or regions in gray plates are level four. Most of these countries are underdeveloped areas with relatively low scientific research capabilities. There have been no articles published in this field within the selected ten-year time span. **Figure 6C** is a diagram of cooperation between countries. Every time there is cooperation between two countries, there will be a connection. It can be seen that the cooperation between China and the United States is close. These two countries also have the most frequent international cooperation at the national level. Finally, there is the total citations of national output (**Figure 6D**). China leads all countries with a citation frequency of 6785, and the gap is huge, followed by the United States and Italy, whose citation frequencies are 2745 and 1280 respectively. China has the highest citation frequency, and it also has the highest number of publications. In both aspects China is in the leading position. However, it cannot be ignored that due to the large number of publications, one possible problem is that although there are many citation frequencies, the average citation frequency is not necessarily leading, and the citation frequency only provides a certain reference basis for judging the extent of a country’s contribution.

**Table 6:** Corresponding authors and collaborations in different countries.

Country	Articles	SCP	MCP	Freq	MCP_Ratio
CHINA	422	390	32	0.46	0.076
USA	114	83	31	0.124	0.272
ITALY	57	46	11	0.062	0.193
JAPAN	40	32	8	0.044	0.2
GERMANY	38	22	16	0.041	0.421
INDIA	21	15	6	0.023	0.286
KOREA	20	16	4	0.022	0.2
POLAND	18	11	7	0.02	0.389
UNITED KINGDOM	15	8	7	0.016	0.467
FRANCE	14	12	2	0.015	0.143

### Keyword analysis

The keywords for our study were sourced from the Web of Science Core Collection, which includes author-provided keywords and ‘Keywords Plus’—terms from cited references’ titles. This ensures a wide-ranging representation of central themes in osteosarcoma metabolomics research.

**Figure 7:**
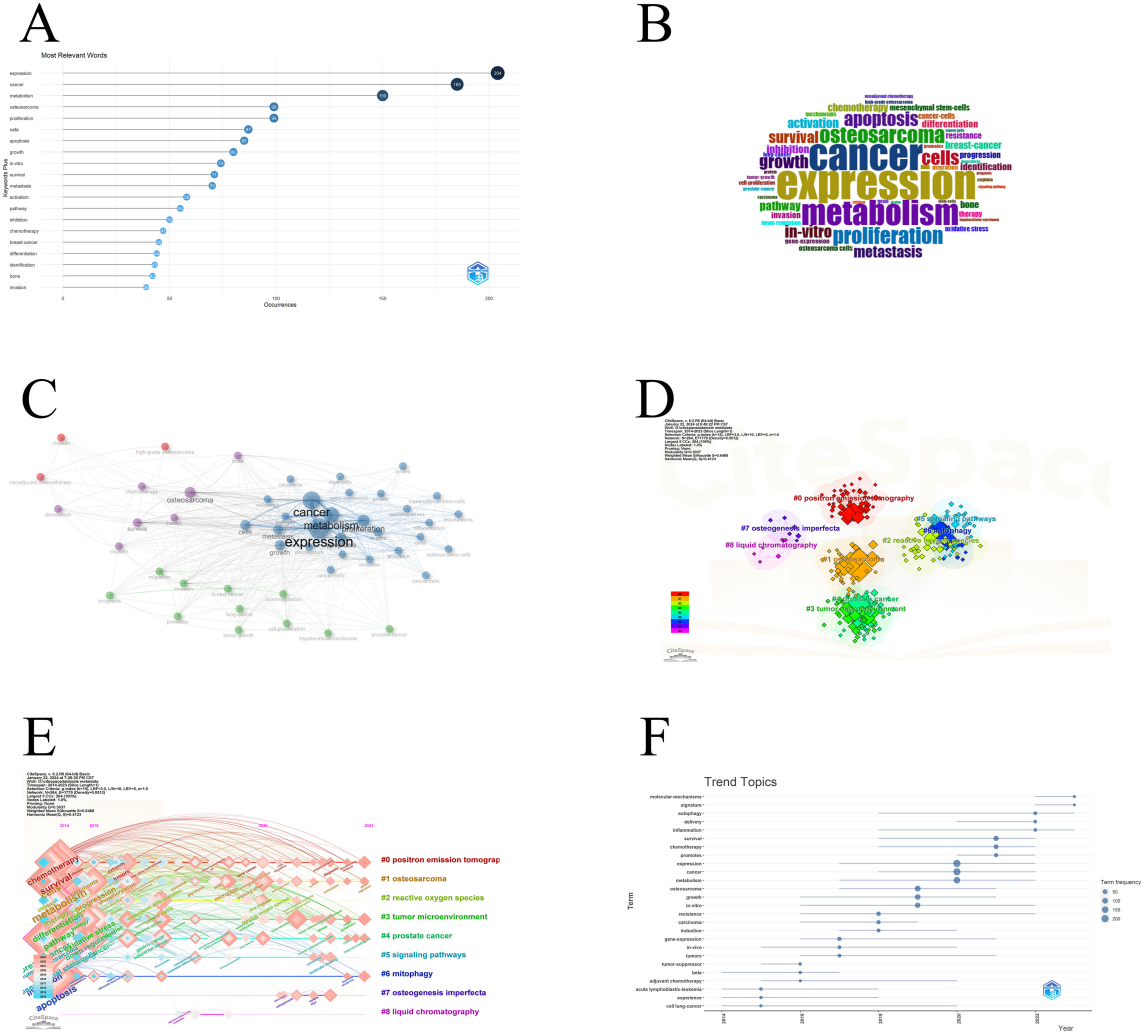
Keyword information: **(A)** Keyword frequency ranking; **(B)** Keyword word cloud diagram; **(C)** Keyword co-occurrence network; **(D)** Keyword clustering; **(E)** Timeline diagram of clustering; **(F)** trending topics.

Analyzing keywords is of great significance, as it helps us discover the research hot spots of scientific researchers in the past ten years and speculate on the problems and research progress in this field. Based on the research hot spots in recent years, the next research direction can also be predicted. **Figure 7A** shows the 15 most relevant keywords in the past ten years and their frequency of occurrence. These keywords are presented in the form of a word cloud as shown in **Figure 7B**. expression is the keyword that has received the most attention in the past decade, which may mean that the expression of certain osteosarcoma metabolism-related genes is very important for this disease. According to the explosive word list (**Table 7**), we can find explosive words in different years. These words are active in researchers’ articles in different years. For example, gene expression was a research hotspot from 2014 to 2017, with an intensity of 4.95. The higher it is, the more relevant research there is in the same year. Tumor microenvironment, mutations, and inhibitors have been active since 2021 until now. These three keywords may be closely related to the next research trends. **Figure 7C** is a keyword co-occurrence network. If two keywords appear together in an article, there will be a connection. The thicker the connection, the closer the connection between the two keywords. The more connected keywords are with the more relevant the other keywords are. The three keywords of cancer, metabolism, and expressions are highly correlated. According to the algorithm of bibliometrix, these keywords are roughly divided into four categories, which are replaced by four colors. The red cluster represents treatment ideas for malignant osteosarcoma in children; the green cluster represents the associations that exist between osteosarcoma and other cancers; the purple cluster represents metabolism-related treatments for osteosarcoma and their effects; and the blue cluster represents expression of related genes in osteosarcoma. These keywords are closely related in their respective categories. We used citespace to conduct a more detailed cluster analysis of keywords (**Figure 7D**). They are:

**Table 7:** Top 22 keywords with the strongest citation bursts.

Keywords	Year	Strength	Begin	End	2014 - 2023
gene expression	2014	4.95	2014	2017	▃▃▃▃▂▂▂▂▂▂
acute lymphoblastic leukemia	2014	3.49	2014	2015	▃▃▂▂▂▂▂▂▂▂
overexpression	2014	2.83	2014	2016	▃▃▃▂▂▂▂▂▂▂
death	2014	2.46	2014	2015	▃▃▂▂▂▂▂▂▂▂
carcinoma	2015	4.58	2015	2019	▂▃▃▃▃▃▂▂▂▂
tumor suppressor	2015	4.52	2015	2016	▂▃▃▂▂▂▂▂▂▂
*in vivo*	2015	3.6	2015	2017	▂▃▃▃▂▂▂▂▂▂
phosphorylation	2016	3.39	2016	2017	▂▂▃▃▂▂▂▂▂▂
tumors	2016	3.23	2016	2017	▂▂▃▃▂▂▂▂▂▂
induction	2016	3.22	2016	2018	▂▂▃▃▃▂▂▂▂▂
extremity	2016	2.65	2016	2018	▂▂▃▃▃▂▂▂▂▂
osteogenic sarcoma	2017	3.6	2017	2018	▂▂▂▃▃▂▂▂▂▂
gene	2017	3.48	2017	2019	▂▂▂▃▃▃▂▂▂▂
cancer cells	2015	3.21	2017	2019	▂▂▂▃▃▃▂▂▂▂
colorectal cancer	2017	2.91	2017	2019	▂▂▂▃▃▃▂▂▂▂
positron emission tomography	2020	3.7	2020	2021	▂▂▂▂▂▂▃▃▂▂
reactive oxygen species	2020	3.23	2020	2021	▂▂▂▂▂▂▃▃▂▂
promotes	2020	3.23	2020	2021	▂▂▂▂▂▂▃▃▂▂
protein	2016	2.54	2020	2021	▂▂▂▂▂▂▃▃▂▂
tumor microenvironment	2021	5.09	2021	2023	▂▂▂▂▂▂▂▃▃▃
mutations	2021	2.57	2021	2023	▂▂▂▂▂▂▂▃▃▃
inhibitor	2021	2.57	2021	2023	▂▂▂▂▂▂▂▃▃▃

0 positron emission tomography

1 osteosarcoma

2 reactive oxygen species

3 tumor microenvironment

4 prostate cancer

5 signaling pathways

6 mitophagy

7 osteogenesis imperfecta

8 liquid chromatography

Distributing these clusters according to the time axis can explore the active status of each cluster in each time period (**Figure 7E**). The large nodes of the first seven clusters all started in 2014, which may have been a lot of research on these nodes in 2014 or before. The two clusters #7 and #8 appear for a shorter time. There are five clusters that continue to this day and have continuing trends. They are #0, #1, #3, #4, and #6. The theme trends also have certain reference significance. **Figure 7F** has appeared in the past ten years. Trend topics, these topics have persisted for a period of time, and some even continue to the present, such as molecular-mechanisms, signature, autophagy, and inflammation.

### Citation analysis

We collected the ten most frequently cited documents in this field and explored what theoretical foundations these documents provide, which can help us understand important information in this field. The collected results are presented in **Table 8**. The most frequently cited reference is by Michael S. Lsakoff, Stefan S. Bielack et al., who discuss biology, preclinical and clinical trial efforts and future international collaboration strategies with the aim of improving the treatment of cancer patients. Treatment results (45). This article became an explosive citation in this field between 2016 and 2020, reflecting the importance of this article. **Table 9** displays burst citations over the years. Examining the temporal characteristics of citations in these articles can determine the high interest in this field at a specific time. One of the earliest Breakout citations was by Daniela Caronia, Ana Patiño-Garcia, et al., who investigated whether common germline polymorphisms in chemotherapeutic transporter and metabolic pathway genes for drugs used in standard osteosarcoma treatments could predict and Correlations between four SNPs in two ATP-binding cassettes and overall survival were found Caronia (46). Anja Luetke, Paul A Meyers, et al. discuss the treatment experience of systemic osteosarcoma (47). Outbreak Citation in Recent Years Jeremy S Whelan, Lara E Davis discuss the treatment of osteosarcoma, chondrosarcoma and chordoma (48). Stefan S Bielack, Jeremy Whelan and others explore survival and prognosis in osteosarcoma (49). In addition, Ingrid Lilienthal and Nikolas Herold summarized the treatment strategies of osteosarcoma in their review, providing a reference for rational treatment (50).

**Table 8:** Top ten lists of citations in the field of osteosarcoma metabolomics.

Cited References	Citations
ISAKOFF MS, 2015, J CLIN ONCOL, V33, P3029, DOI 10.1200/JCO.2014.59.4895	(40)
KANSARA M, 2014, NAT REV CANCER, V14, P722, DOI 10.1038/NRC3838	(41)
LUETKE A, 2014, CANCER TREAT REV, V40, P523, DOI 10.1016/J.CTRV.2013.11.006	(42)
BIELACK SS, 2002, J CLIN ONCOL, V20, P776, DOI 10.1200/JCO.20.3.776	(43)
HANAHAN D, 2011, CELL, V144, P646, DOI 10.1016/J.CELL.2011.02.013	(43)
MIRABELLO L, 2009, CANCER-AM CANCER SOC, V115, P1531, DOI 10.1002/CNCR.24121	(43)
OTTAVIANI G, 2009, CANCER TREAT RES, V152, P3, DOI 10.1007/978-1-4419-0284-9_1	(44)
HEIDEN MGV, 2009, SCIENCE, V324, P1029, DOI 10.1126/SCIENCE.1160809	(16)
GILL J, 2021, NAT REV CLIN ONCOL, V18, P609, DOI 10.1038/S41571-021-00519-8	(35)
HARRISON DJ, 2018, EXPERT REV ANTICANC, V18, P39, DOI 10.1080/14737140.2018.1413939	(34)

**Table 9:** Top 25 references with the strongest citation bursts.

References	Year	Strength	Begin	End	2014 - 2023
Hanahan D, 2011, CELL, V144, P646, DOI 10.1016/j.cell.2011.02.013	2011	4.8	2014	2016	▃▃▃▂▂▂▂▂▂▂
Caronia D, 2011, PLOS ONE, V6, P0, DOI 10.1371/journal.pone.0026091	2011	4.26	2014	2016	▃▃▃▂▂▂▂▂▂▂
Luetke A, 2014, CANCER TREAT REV, V40, P523, DOI 10.1016/j.ctrv.2013.11.006	2014	11.81	2015	2019	▂▃▃▃▃▃▂▂▂▂
Kansara M, 2014, NAT REV CANCER, V14, P722, DOI 10.1038/nrc3838	2014	6.5	2015	2018	▂▃▃▃▃▂▂▂▂▂
Jones KB, 2012, CANCER RES, V72, P1865, DOI 10.1158/0008-5472.CAN-11-2663	2012	4.15	2015	2017	▂▃▃▃▂▂▂▂▂▂
Anninga JK, 2011, EUR J CANCER, V47, P2431, DOI 10.1016/j.ejca.2011.05.030	2011	3.49	2015	2016	▂▃▃▂▂▂▂▂▂▂
He JP, 2014, ASIAN PAC J CANCER P, V15, P5967, DOI 10.7314/APJCP.2014.15.15.5967	2014	3.1	2015	2017	▂▃▃▃▂▂▂▂▂▂
Duan ZF, 2011, MOL CANCER THER, V10, P1337, DOI 10.1158/1535-7163.MCT-11-0096	2011	2.91	2015	2016	▂▃▃▂▂▂▂▂▂▂
Broadhead Matthew L, 2011, SARCOMA, V2011, P959248, DOI 10.1155/2011/959248	2011	2.91	2015	2016	▂▃▃▂▂▂▂▂▂▂
Namlos HM, 2012, PLOS ONE, V7, P0, DOI 10.1371/journal.pone.0048086	2012	2.58	2015	2017	▂▃▃▃▂▂▂▂▂▂
Isakoff MS, 2015, J CLIN ONCOL, V33, P3029, DOI 10.1200/JCO.2014.59.4895	2015	10.13	2016	2020	▂▂▃▃▃▃▃▂▂▂
Bonuccelli G, 2014, ONCOTARGET, V5, P7575, DOI 10.18632/oncotarget.2243	2014	4.46	2016	2019	▂▂▃▃▃▃▂▂▂▂
Chen X, 2014, CELL REP, V7, P104, DOI 10.1016/j.celrep.2014.03.003	2014	2.86	2016	2017	▂▂▃▃▂▂▂▂▂▂
Ferrari S, 2015, EXPERT OPIN PHARMACO, V16, P2727, DOI 10.1517/14656566.2015.1102226	2015	2.57	2017	2020	▂▂▂▃▃▃▃▂▂▂
Baglio SR, 2017, CLIN CANCER RES, V23, P3721, DOI 10.1158/1078-0432.CCR-16-2726	2017	3.48	2018	2021	▂▂▂▂▃▃▃▃▂▂
Durfee RA, 2016, RHEUMATOL THER, V3, P221, DOI 10.1007/s40744-016-0046-y	2016	3.45	2018	2020	▂▂▂▂▃▃▃▂▂▂
Lindsey BA, 2017, RHEUMATOL THER, V4, P25, DOI 10.1007/s40744-016-0050-2	2017	3.24	2019	2021	▂▂▂▂▂▃▃▃▂▂
Pavlova NN, 2016, CELL METAB, V23, P27, DOI 10.1016/j.cmet.2015.12.006	2016	4.29	2020	2021	▂▂▂▂▂▂▃▃▂▂
Gianferante DM, 2017, NAT REV ENDOCRINOL, V13, P480, DOI 10.1038/nrendo.2017.16	2017	3.39	2020	2023	▂▂▂▂▂▂▃▃▃▃
Ren L, 2017, ONCOTARGET, V8, P38541, DOI 10.18632/oncotarget.15872	2017	3.14	2020	2021	▂▂▂▂▂▂▃▃▂▂
Whelan JS, 2018, J CLIN ONCOL, V36, P188, DOI 10.1200/JCO.2017.75.1743	2018	4.33	2021	2023	▂▂▂▂▂▂▂▃▃▃
Smeland S, 2019, EUR J CANCER, V109, P36, DOI 10.1016/j.ejca.2018.11.027	2019	3.6	2021	2023	▂▂▂▂▂▂▂▃▃▃
Lilienthal I, 2020, INT J MOL SCI, V21, P0, DOI 10.3390/ijms21186885	2020	2.58	2021	2023	▂▂▂▂▂▂▂▃▃▃
Wu CC, 2020, NAT COMMUN, V11, P0, DOI 10.1038/s41467-020-14646-w	2020	2.51	2021	2023	▂▂▂▂▂▂▂▃▃▃
Ren L, 2020, CANCER METAB, V8, P0, DOI 10.1186/s40170-020-0209-8	2020	2.51	2021	2023	▂▂▂▂▂▂▂▃▃▃

## Discussion

Tumors have always been a problem that plagues mankind, and osteosarcoma is no different. Osteosarcoma is a malignant tumor that originates from bone tissue and is highly invasive. It usually occurs in children and adolescents (51). Treatment of osteosarcoma is a challenge, but there are several treatment options available for osteosarcoma. Examples include surgical treatment, chemotherapy, radiation therapy, and targeted therapy. Surgery is the cornerstone of osteosarcoma treatment, and the lesion is removed surgically. Limb-sparing surgery can be performed to avoid amputation when the lesion is confined, but may be necessary when the lesion is more extensive. Chemotherapy is indispensable in the treatment of osteosarcoma. Chemotherapy before surgery can reduce the size of the tumor and create conditions for surgery, and chemotherapy after surgery can remove undetected metastatic cancer cells. Commonly used drugs include cisplatin, adriamycin and methotrexate. Radiation therapy also plays a role in the treatment of osteosarcoma, which is usually used when the tumor cannot be removed by surgery or recurs. Targeted therapy is a relatively new treatment. It uses drugs such as IGF-1R or mTOR to inhibit tumor growth in order to achieve a therapeutic effect. Immunotherapy is also being developed, which aims to induce the body’s immune system to recognize and attack cancer cells. In conclusion, in addition to traditional treatment options, more and more new treatment options are being developed (1).

Osteosarcoma is defined as a rare disease, and research on osteosarcoma has been ongoing, but due to the small number of patients, less research has been conducted on this field. Not only is there a lack of researchers, but limited by theories and technology, research in this area is also difficult to carry out. With the development of medical technology, more and more advanced instruments are used in medical research, and more relevant theories have been discovered. Benefiting from these, research in the field of osteosarcoma metabolism has begun to develop rapidly, and more and more metabolic pathways are being explored, which also means that there will be more therapeutic options to try.

Studying the metabolic processes of osteosarcoma can help understand the development of this cancer. Metabolic abnormalities in osteosarcoma cells may cause them to proliferate excessively and evade immune system attack (44). Researchers study the metabolic pathways of osteosarcoma, which may lead to new treatment strategies and drugs (52).

Bibliometrics is the study of quantitative analysis methods of scientific literature and academic output. Bibliometrics is widely used. It can use statistical methods and mathematical tools to analyze and evaluate the characteristics, trends and influence of documents in terms of academic output, citations, cooperation relationships, etc. In this study, we used bibliometrics to analyze the products of osteosarcoma metabolism research from 2014 to 2023. We used cite space and bibliometrix to analyze 917 documents, and systematically analyzed institutions, journals, countries or regions, authors, keywords, and citations. Through these analyses, we can speculate on the current research status and future trends in this field.

### Knowledge base

Judging from the number of publications in this field, there is an overall upward trend, but there has been a downward trend since 2022. The citations of literature in this field have declined significantly since 2019, largely attributable to a series of pioneering studies that introduced novel therapeutic approaches and advanced molecular insights into osteosarcoma. These studies not only provided innovative solutions, such as the development of liposome-encapsulated curcumin-loaded 3D printed scaffolds and the use of black phosphorus for treatment (43, 53), but also delved into the intricate molecular mechanisms underpinning cancer progression, such as m6A methylation and its regulatory roles (54). The interdisciplinary methodologies employed, combining elements from genetic engineering, material science, and biochemistry, have paved the way for potential clinical translations, directly impacting patient care and treatment protocols (55). Such contributions are critically important not only for their immediate academic and clinical implications but also for their broader relevance to public health, given the severe impact of osteosarcoma on younger populations. The high citation rates of these publications underscore their significant influence on the field, driving forward the boundaries of research and offering new avenues for improving outcomes in osteosarcoma treatment. After this, the quality of the articles began to decline, and researchers may need to look for new ideas to conduct more meaningful experiments.

In terms of institutions, Shanghai Jiao Tong University ranks first in the number of publications. Shanghai Jiao Tong University is a famous comprehensive university in China, located in Shanghai, China. Shanghai Jiao Tong University has a long history, which can be traced back to the 1890s (https://www.sjtu.edu.cn). Their research results include that miR-22 effectively inhibits tumor growth and metastasis by targeting and inhibiting ATP citrate lyase (ACLY), providing potential therapeutic benefits for osteosarcoma, prostate cancer, cervical cancer, and lung cancer (56). M6A alpha Base transferase METTL3 enhances osteosarcoma progression by increasing m6A methylation of LEF1 mRNA, thereby activating the Wnt/β-catenin signaling pathway and promoting cancer cell proliferation, migration, and invasion (54). In addition to Shanghai Jiao Tong University, there are five institutions originating from China, namely Central South University, Zhejiang University, Nanjing Medical University, Chinese Academy of Sciences and Fudan University. From this information, it can be seen that China’s interest in this field is higher than that of other countries. The fourth-ranked University of Texas System is a public university located in Texas, USA(https://www.utsystem.edu). Founded in 1876, it is a university with a high reputation in many aspects. Contributions to medicine in the University of Texas System usually originate from its medical and biological research institutions. Among them, the University of Texas MD Anderson Cancer Center has a high reputation in cancer research, and most of its contributions in the field of osteosarcoma metabolism come from this health science center.

The top three core journals among the journals are INTERNATIONAL JOURNAL OF MOLECULAR SCIENCES, ONCOTARGET, and FRONTIERS IN ONCOLOGY. They are all important journals in the field of biology. The International Journal of Molecular Sciences is an open international academic journal that focuses on research in the molecular field, such as molecular biology, molecular physics, etc. Research on the molecular mechanisms of osteosarcoma metabolism will be accepted by this journal. Oncotarget’s focus is mainly on oncology research. In addition to oncology, the topics involved include neuroscience, endocrinology, cardiovascular disease and other disciplines. As a type of malignant tumor, related research on osteosarcoma can also be published in this journal. Frontiers in Oncology is a sub-journal of Frontiers. This sub-journal also focuses on the field of oncology. This journal is more inclined to multidisciplinary discussions and covers various branches of oncology. In this journal, you can see research on the metabolism of osteosarcoma. Recent advances in molecular mechanism research, emerging treatment options, and cancer metabolism.

Among the authors, two authors whose identities have been clearly identified have more than 10 publications, namely Avbet, Soflia (12), and Baldini, Nicola (10). These two authors have a close cooperative relationship. Avbet, Soflia and Baldini, Nicola are both from the Department of Biomedicine and Neuromotor Sciences of the University of Bologna, Italy. Their academic focus is on applied medical technology and methodology. Avbet, Soflia is a senior assistant professor with an extensive educational background including a degree in medical biotechnology, a PhD in the same field, a master’s degree in cell and molecular biology, etc. (https://www.unibo.it/sitoweb/sofia.avnet3/cv-en). Baldini, Nicola is a full professor and not only that, he works as an orthopedist and oncologist at the Istituto Ortopedico Rizzoli (https://www.unibo.it/sitoweb/nicola.baldini5/en). The two of them had many collaborative relationships in the past ten years, and the most cited article during the period was jointly published by multiple authors, titled The human tumor microbiome is composed of tumor type-specific intracellular bacteria (42). This study provided a comprehensive analysis of tumor microbiome, Includes 7 types of cancer. Study finds each tumor type has unique microbial composition, with breast cancer in particular having a rich and diverse microbiome. The most recent article was published in cancers on February 21, 2023, and was also published by two people. This article studied the contribution of mitochondrial activity to doxorubicin resistance in osteosarcoma cells. The study pointed out that compared with sensitive cells, doxorubicin-resistant clones showed higher activity and lower oxygen-dependent metabolism. and had significantly reduced mitochondrial membrane potential, mitochondrial mass, and reactive oxygen species (2) production. Reduced TFAM gene expression in drug-resistant cells is often associated with mitochondrial biogenesis, and combining doxorubicin (a chemotherapy drug) with quercetin (a known inducer of mitochondrial biogenesis) Drug-resistant osteosarcoma cells can be resensitized to doxorubicin (57). In addition to these two authors, there are still a few authors who have been conducting research in this field for a long time, but most of them have low scientific output. Governments should increase funding or formulate relevant policies to encourage researchers to conduct more valuable research.

Among countries or regions, China is the main leader. Our study reveals China’s prominent role in advancing osteosarcoma metabolomics research and demonstrates China’s strategic investment in cancer research and innovation. There are the largest number of corresponding authors from China, China has the largest scientific output, and China has the largest total number of citations. But most authors in China are not good at international cooperation, which is a negative phenomenon. The analysis shows a relatively isolated research landscape with limited international collaboration. Strengthening global research partnerships can accelerate translation of metabolomics findings into clinical practice. International cooperation among academics is conducive to promoting information exchange among countries and accelerating progress in this field. There are many reasons for failure to cooperate, such as distance, policy, scientific research level, etc. Judging from the status of the authors’ publications, we speculate that some authors in China have low scientific research levels and have not reached the threshold for international cooperation. This is reflected in a large number of low-publishing authors. In general, China has many authors, but the quality of articles still needs to be improved. We recommend that researchers continue to conduct more valuable research to improve the overall quality of articles, and that the government increases investment to help researchers conduct research by formulating relevant policies.

To promote broader international collaboration and enhance dissemination of research results in the field of osteosarcoma metabolomics, we propose the establishment of a global alliance. The organization will provide a platform for researchers from various countries to share their results, discuss methods and achieve breakthroughs in the treatment and understanding of osteosarcoma at a faster pace by promoting open dialogue and collaboration.

In the realm of keywords, “express” has consistently garnered attention. Commonly referring to gene expression, this focus is sustained due to ongoing research into genes related to the metabolism of osteosarcoma. For instance, a review published in 2023 revisited the miR-30 gene family prior to that year, with previous studies indicating that low expression of miR-30c correlates with higher malignancy and shorter survival in osteosarcoma (41). Other genes within the same family play crucial roles in bone metabolism. Another review from 2020 discussed the expression of autocrine motility factors in bone tumors, finding that these factors and their receptors collectively alter the bone microenvironment (58). Additionally, autocrine motility factors are involved in processes such as glycolysis, gluconeogenesis, and protein degradation. More recent research has shown that patients with osteosarcoma, rhabdomyosarcoma, and angiosarcoma tend to express higher levels of AMF, while patients with multiple myeloma show higher expression of AMFR. A 2019 review summarized studies on low-density lipoprotein in metabolism, noting that during the study period, LRP5 was proven to play a role in osteosarcoma and in non-cancer conditions such as the chondrocytic subtype of prostate cancer and osteoporosis (32). Furthermore, a 2021 review revisited the relationship between LOX-1 and cancer, discussing the correlation between tumors, metabolic disorders, and new therapeutic strategies (40). The authors highlighted the role of the LOX-1 receptor in various cancers, such as glioblastoma and osteosarcoma, and evidence of its interaction with the WNT/APC/beta-catenin signaling pathway. They also mentioned that targeting LOX-1 to inhibit angiogenesis and metastasis represents a promising anti-cancer strategy.

### Research trends

**Figure 7F** shows the evolution of research topics from 2014 to 2023. It is clear that some topics have maintained a stable presence in academic discussions over the years, with “gene expression” continuing to attract attention, indicating continued research interest and potential for continued breakthroughs in this area. On the other hand, terms such as “autophagy” and “transmission” have significantly increased in frequency, suggesting that these fields have gained momentum, possibly due to new discoveries or technological developments that make research in these areas more feasible or relevant of. Node sizes corresponding to “inflammation” and “metabolism” also grew significantly and remained prominent around 2018, reflecting the growing awareness of the role of inflammation in disease and the surge in metabolism-related research, likely due to for their importance in diseases such as cancer. Notably, “osteosarcoma” has peaked, reflecting a specific period of concentrated research effort, which may be associated with important publications or clinical trials reporting significant findings. In contrast, topics such as “acute lymphoblastic leukemia” and “cellular lung cancer” appear less frequently, which may represent a shift or maturation of research focus in these areas. Overall, the focus of this research area continues to evolve under the influence of technological advances, clinical needs, and scientific discoveries.

Based on the keyword clustering, trending topics and explosive words that continue to this day, we can speculate on the research hot spots in the short term. It can be roughly divided into three aspects: tumor microenvironment, molecular mechanism and autophagy, targeted therapy and inhibitors.

#### Tumor microenvironment

The tumor microenvironment is the environment around tumor cells, including various cells and extracellular components for the occurrence and development of tumor cells. Cells include immune cells, vascular endothelial cells, fibroblasts, etc. These cells play an important role in tumor development, treatment and tumor cell metastasis. Extracellular components include growth factors, chemical factors, pro-inflammatory cytokines, tumor necrosis factor, etc., which can all affect tumor cells. Studying the tumor microenvironment helps to understand the development of cancer, explore new treatment strategies, immune evasion mechanisms and drug resistance of cancer cells (59, 60). In July 2023, Qiu Xinzhu, He Hongbo and others systematically correlated MYBL2 with immune signatures in the pan-cancer tumor microenvironment, and used their risk score to predict the prognostic ability of osteosarcoma. They pointed out that MYBL2 regulates the proliferation development and immune infiltration of osteosarcoma and pan-cancer, and MYBL2 can be used as a potential marker in the osteosarcoma microenvironment to predict prognosis (61). An article published in January 2024 used single-cell sequencing data to identify exhausted T cells in the osteosarcoma microenvironment, investigated the role of T cell depletion in the osteosarcoma microenvironment, and provided insights into osteosarcoma development. New insights into mechanisms and their therapeutic strategies (62). In the same month, Wang Yang, Zhou Xueru and others published an article on the use of capsaicin in the adjuvant treatment of osteosarcoma. The study pointed out that capsaicin can induce ferroptosis, relieve hypoxia, destroy redox homeostasis, and enhance the effect of photodynamic therapy (63).

#### Molecular mechanisms and autophagy

The molecular mechanisms related to osteosarcoma metabolism mainly involve signal transduction pathways, gene expression regulation, and cell proliferation and apoptosis mechanisms. Research mainly focuses on the occurrence, development and treatment response mechanisms of osteosarcoma, especially the key molecules and pathways that lead to tumor growth and metastasis. Autophagy is an important process that maintains cellular homeostasis. This process is complex and involves the removal of damaged proteins and organelles. In osteosarcoma, autophagy may play a dual role, that is, it can induce cell death and maintain the survival of tumor cells. Understanding molecular mechanisms and autophagy can provide insights into the mechanisms of cancer and can also help develop new treatment strategies. For example, the cancer-promoting protein IF1 helps tumor cell growth by promoting mitochondrial renewal and energy conservation (64). Sergio Almansa-Gomez, Francisco Prieto-Ruiz and others made a review on the regulation of autophagy in osteosarcoma. They believed that many results have been achieved in the regulation of autophagy in osteosarcoma in the past, but future research still needs to elucidate the role of autophagy. Molecular mechanisms and their relationship to osteosarcoma (65).

#### Targeted therapies and inhibitors

The identification of new molecular targets, such as specific mutations or pathways active in osteosarcoma cells, is a key area. The development of inhibitors that target these molecules or pathways may provide new treatment options for osteosarcoma. CAR-T cell therapy, which has attracted much attention since its discovery, involves targeting tumor antigens and releasing immune factors. This technology has made significant progress in hematological malignancies, but is subject to many limitations in osteosarcoma. Nonetheless, this is still a potential treatment and research is ongoing (66). A study published in January 2024 showed that the use of a first-in-class RNA polymerase mitochondrial inhibitor IMT1 can inhibit the survival, proliferation, migration and other activities of osteosarcoma cells (67). Another study in the same month verified that inhibiting stearoyl-CoA desaturase can impair the proliferation and invasion of osteosarcoma and become a therapeutic target for new drugs (68).

In the context of tumors, metabolomics research continues, and significant research results have been achieved in various types of tumors. Even one metabolic pathway can be mapped to multiple types of tumors, highlighting the interconnected nature of metabolic processes across different cancers. This cross-tumor applicability suggests that discoveries in osteosarcoma can often provide valuable insights into others, potentially leading to broader therapeutic applications. For instance, alterations in glycolysis and lipid metabolism pathways, frequently observed in several cancers, have inspired new approaches to treatment that target these shared metabolic changes. Such findings underscore the importance of metabolomics not only in understanding the unique metabolic fingerprints of each cancer type but also in uncovering universal targets that could lead to more effective, multi-faceted treatment regimens.

### Limitation

First, due to software limitations, we were unable to cross-validate with other databases, which may have led to biased findings. Second, it’s important to acknowledge that the results of the bibliometric analysis could be influenced by the authors’ comprehension of the subject matter and certain subjective factors. Third, bibliometric analyses have a certain timeliness due to the dynamic nature of database updates.

## Conclusion

Using bibliometric methods, we conducted a comprehensive visual analysis of the field of osteosarcoma metabolism, revealed research hotspots in each period, and predicted future trends. It is noteworthy that China occupies a major leadership position in this field, but most authors from China still need to improve their scientific research standards. There is still much room for collaboration among individual authors, institutions, and countries. At present, it seems that the next research may focus more on the three directions of tumor microenvironment, molecular mechanism and autophagy, as well as targeted therapy and inhibitors.

## Data Availability

The original contributions presented in the study are included in the article/supplementary material, further inquiries can be directed to the corresponding author/s.
